# MicroRNA-4290 suppresses PDK1-mediated glycolysis to enhance the sensitivity of gastric cancer cell to cisplatin

**DOI:** 10.1590/1414-431X20209330

**Published:** 2020-04-17

**Authors:** Yan Qian, Xu Wu, Haixiao Wang, Guowei Hou, Xiao Han, Wei Song

**Affiliations:** 1 Department of Gastric Surgery, The Affiliated Huaian No.1 People's Hospital of Nanjing Medical University, Huai'an, Jiangsu, China; 2 Department of Gastroenterology, The Affiliated Huaian No.1 People's Hospital of Nanjing Medical University, Huai'an, Jiangsu, China

**Keywords:** miR-4290, PDK1, Cisplatin, Chemoresistance, Gastric cancer

## Abstract

The development of chemotherapy resistance significantly impairs the efficiency of chemotherapy, but the underlying mechanisms of chemotherapy resistance in gastric cancer (GC) are complicated and still need to be further explored. Here, we aimed to reveal the effects of miR-4290/PDK1 (pyruvate dehydrogenase kinase 1) axis on chemotherapy resistance of GC *in vitro*. The expression patterns of miR-4290 in GC tissues and cell lines were determined by real-time quantitative PCR. Kaplan-Meier was used to assess the relationship between miR-4290 expression levels and patients' overall survival. CCK-8 and flow cytometry technologies were applied to detect cell proliferation and apoptosis. The luciferase gene reporter assay was used to evaluate the interaction between miR-4290 and PDK1. miR-4290 was lowly expressed in GC tissues and cell lines, which was closely associated with the shorter overall survival of GC patients. miR-4290 mimics significantly inhibited cell proliferation and induced cell apoptosis, as well as induced a significant reduction in the expression of PDK1. Moreover, miR-4290 significantly inhibited glycolysis and decreased the IC50 value to cisplatin in SGC7901 cells, whereas these effects were abolished and cell apoptosis was promoted when PDK1 was overexpressed. In conclusion, this study revealed that miR-4290 suppressed PDK1-mediated glycolysis to enhance the sensitivity of GC cells to cisplatin.

## Introduction

Gastric cancer (GC) is the most common solid tumor originating from the digestive system and is a major reason for cancer-related mortality in the world (1). The 5-year survival rate for GC patients is only 30∼50% due to the high recurrence and metastasis rate (2). The standard treatment strategy for GC without distant metastasis is surgery resection, and chemotherapy is a good supplement approach because it can efficaciously prevent its metastasis and recurrence (3). However, the occurrence and development of chemotherapy resistance seriously impair the efficiency of chemotherapy (4). Therefore, the underlying mechanisms of chemotherapy resistance in GC need to be further explored.

Cancer cells undergo glycolysis in the presence of oxygen, which is called the Warburg effect. In contrast, the non-cancer cells can flexibly change based on molecular oxygen availability. Accumulated evidence has demonstrated that the aberrant activation of glycolysis plays a crucial role in many kinds of diseases via various mechanisms, including the induction of cancer chemotherapy resistance (5–7). Pyruvate dehydrogenase kinase 1 (PDK1) is an important glycolytic enzyme that can phosphorylate and inactivate pyruvate dehydrogenase and thereby suppress pyruvate oxidation (8). As a glycolytic enzyme, PDK1 has been identified to be closely associated with cancer cell proliferation, metastasis, and chemotherapy resistance (9–11). For instance, Qin et al. (12) found that PDK1 inhibitor dichloroacetophenone significantly inhibited acute myeloid leukemia cell proliferation and autophagy, and induced apoptosis. These findings suggest that PDK1 might be a potent target for reversing chemotherapy resistance.

MicroRNA (miRNA) is a class of non-coding RNAs with 18–22 nucleotides in length, which can repress the biological function of target genes via a direct binding to the 3′ untranslated region (UTR) of the target gene (13). miRNAs are widely expressed in living individuals and have been identified to play an important role in the pathogenesis of many kinds of diseases, including the carcinogenesis of GC (14–16). Noticeably, miRNAs are strongly implicated in the chemotherapy resistance of GC. For example, Gong et al. (17) found that the expression of miR-625 was decreased in multidrug resistance compared to that of the chemosensitive counterparts, and overexpression of miR-625 significantly enhanced the sensitivity and induced cell apoptosis in GC. Li et al. (18) demonstrated that miR-200c-3p upregulation reversed drug resistance of human GC via regulating NER-ERCC3/4 pathway. Moreover, Peng et al. (19) reported that enforced expression of miR-494 significantly increased the chemosensitivity of GC cells to doxorubicin, and inhibited cell viability and colony formation ability by targeting phosphodiesterases 4D.

Bioinformatics results showed that PDK1 was a target of miR-4290, indicating that miR-4290 might be involved in the regulation of chemotherapy resistance of GC cells via targeting PDK1. In this study, we aimed to explore the effects of miR-4290/PDK1 axis on the chemotherapy resistance of GC cells *in vitro*.

## Material and Methods

### GC tissues

GC tissues and matched paracancerous non-tumor tissues were obtained from 60 GC patients who received gastrectomy at the Affiliated Huaian No.1 People's Hospital of Nanjing Medical University from June 2015 to June 2017. None of the patients had received radiotherapy or chemotherapy prior to surgery. This study was performed in accordance with the Helsinki Declaration and approved by the Ethical Committee of the institution. MiR-4290 expression level in GC tissues was regarded as “high” when the expression level was higher than the median level, and was regarded as “low” when its expression level was lower than the median level.

### Cell lines and culture

Human gastric mucosa cell line GES-1, human GC cell lines (SGC7901, MKN45, HGC-27), and human embryonic kidney cell line HEK-293 were purchased from Cell Culture Preservation Committee, Chinese Academy of Sciences (China). GES-1 and HEK-293 cells were maintained in Dulbecco's modified Eagle medium (DMEM); SGC7901, MKN45, and HGC-27 cells were grown in RPMI-1640 medium, containing 10% fetal bovine serum (FBS) and 1% penicillin/streptomycin. All the above reagents used in cell culture were purchased from Thermo Fisher Scientific (USA). Cells used in the experiments were obtained following 48 h of culture at 37°C.

### Cell transfection

The mimics and inhibitors used to overexpress and silence miR-4290 in SGC7901/HGC-27 cells, and the negative control vectors (mimics-NC, inhibitor-NC) were generated by GenePharma (China). The plasmid applied to overexpress PDK1 (named as PDK1), and its control vector (vector) were obtained from OriGene (No. NM_002610; China). These vectors were transfected into cells using Lipofectamine 2000 reagent (Invitrogen, USA) according to manufacturer's instructions. Mimics: 5′-UGCCCUCCUUUCUUCCCUC-3′; inhibitors: 5′-GAGGGAAGAAAGGAGGGCA-3′; NC: 5′-CAGUACUUUUGUGUAGUACAA-3′.

### Real-time quantitative PCR (RT-PCR)

Trizol reagent (Thermo Fisher Scientific) was used to extract the total RNA from GC tissues and cells. Then, a SYBR Green PCR kit (Takara Bio Inc., Japan) was applied to detect the expression levels of PDK1 and GAPDH. The expression levels of miR-4290 and U6 were detected by the TaqMan PCR analysis using an All-in-One miRNA qRTPCR detection kit (GeneCopoeia, USA). GAPDH and U6 levels were used to normalize PDK1 and miR-4290 expression, respectively. The relative expression levels of miRNAs and mRNA were calculated by the 2^−ΔΔCT^ method (20). PDK1-forward: 5′- AAGCAGTTCCTGGACTTCGG-3′, PDK1-reverse: 5′-TCTTGCAGGCCATACAGCAT-3′; GAPDH-forward: 5′-CACTAGGCGCTCACTGTTC-3′, GAPDH-reverse: 5′-GAGGGATCTCGCTCCTGGAA-3′.

### Western blotting assay

Total protein was extracted and isolated using RIPA lysis buffer (Beyotime, China) according to the manufacturer's instructions. The protein concentrations of each sample were then determined using a BCA kit (Thermo Fisher Scientific), followed by degeneration at 100°C for 10 min. Equal amount of proteins 20-30 μg) were loaded onto 10% SDS-PAGE and then probed with the indicated primary antibodies at 4°C overnight, including PDK1 (1:1000 dilution, No. ab110025, Abcam, USA), cleaved caspase-3 (1:1000 dilution, No. ab2302, Abcam), total caspase-3 (1:2000 dilution, No. ab13847, Abcam), cleaved PARP (1:1000 dilution, No. ab32064, Abcam), PARP (1:1000 dilution, No. ab74290, Abcam), and GAPDH (1:5000 dilution; No.TA-08, Zhongshanjinqiao Biotechnology Co., China). The membranes were then probed with the corresponding secondary antibodies (Proteintech, China) at room temperature for 1 h. The membranes were incubated with an enhanced chemiluminescence reagent (ECL; Millipore, USA) and the protein expression levels were then measured by the gel imaging instrument (Eberhardzell, Germany) according to the manufacturer’s directions. Protein expression levels were analyzed by the ImageJ software (NIH, USA).

### Luciferase gene reporter assay

The putative binding sites between miR-4290 and PDK1 were predicted using the TargetScan (http://www.targetscan.org/vert_72/) and miRDBs (http://www.mirdb.org/) online softwares. The 3′-UTR of the wild type (WT) or the mutated type (MUT) of PDK1 were cloned into the luciferase gene reporter vector (Promega, USA), and were called PDK1-WT and PDK1-MUT, respectively. HEK-293 cells were seeded in 24-well plates and were co-transfected with PDK1-WT/PDK1-MUT and mimics/mimics-NC using Lipofectamine 2000 reagent (Invitrogen). The luciferase reporter activity was determined using Promega dual-luciferase reporter assay system after 48 h of cell transfection.

### Glucose uptake and lactate production

SGC7901/HGC-27 cells were inoculated in 6-well plates at a density of 1×10^6^ cells/well. Following 24 h of incubation, the culture medium was collected for the measurement of glucose and lactate levels using an SBA-40C Biosensor (Biology Institute of the Shandong Academy of Science, China). Glucose uptake or lactate production was quantified based on the concentration difference between the cell culture medium and fresh medium without cells.

### Intracellular ATP contents

The level of intracellular ATP was detected using an ATP Colorimetric/Fluorometric Assay kit (Sigma-Aldrich, USA) according to the manufacturer's instructions.

### Measurement of the sensitivity of SGC7901/HGC-27 cells to cisplatin

The drug sensitivity was measured as previously described (21). In brief, after 48 h of cell transfection, 5×10^3^ SGC7901/HGC-27 cells were plated into 96-well plates and administered with different concentrations of cisplatin and incubated for 48 h at 37°C. Then, 20 μg 3-,5-dimethyl-2-thiazolyl)-2,5-diphenyl-2H tetrazolium bromide (MTT) (Merck KGaA, Germany) solution diluted in 200 μL cell culture medium was added into each well and incubated for another 4 h, followed by incubation with 150 μL dimethyl sulfoxide (DMSO) for 10 min. Absorbance at 490 nm was measured using a spectrophotometer (Thermo Fisher Scientific, USA). The concentration at 50% inhibition of growth (IC50) was determined using the relative survival curve.

### Cell proliferation detection

Cell proliferation was detected using the MTT assay (Merck) in accordance with the instructions. In brief, 3×10^3^ SGC7901/HGC-27 cells were seeded into 96-well plates with 200 μL of growth medium. Cell culture medium was replaced with fresh medium every 2 days. Next, cells were incubated with 20 μg of MTT diluted in 200 μL of cell culture medium at 37°C for 4 h after 24, 48, 72, or 96 h of transfection, followed by incubation with 150 μL of dimethyl sulfoxide (DMSO) for 10 min. The absorbance was measured at 490 nm.

### Flow cytometry assay

Cell apoptotic rates were detected by the FITC labelled Annexin V Apoptosis Detection kit (BD Biosciences, USA). After 48 h of cell transfection, SGC7901/HGC-27 cells were harvested and washed with PBS. Next, cells were probed with 100 μL of 1× binding buffer solution and 5 μL of Annexin V and 5 μL of PI solutions were added in the dark for 15 min. After washing with 1× binding buffer, the cells were resuspended and detected using a flow cytometer (BD Biosciences). Cell apoptotic rates were determined by FlowJo 7.6 software (BD Biosciences).

### Statistical analysis

All experiments were performed three times. Data are reported as means±SD. Statistical analyses were performed using the GraphPad Prism 6 software (USA). Student's *t*-test was used for comparisons between two groups and one-way analysis of variance (one-way ANOVA) followed by Tukey's test was used for multiple groups comparisons. Chi-squared test was used for comparisons in Table 1. P<0.05 was considered to be statistically significant.

## Results

### Expression of miR-4290 was decreased in GC tissues and cells and predicted an advanced clinical process and poor prognosis

**Figure 1:**
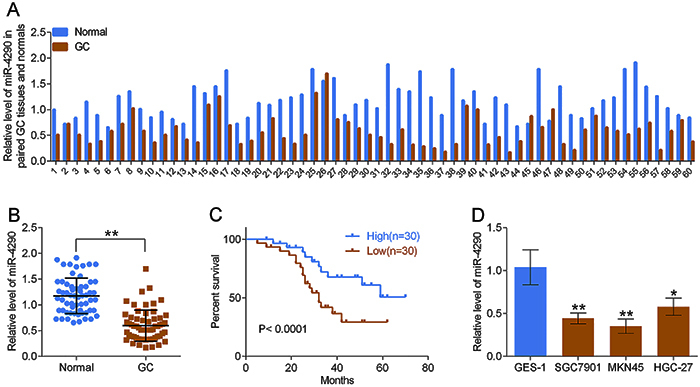
miR-4290 was lowly expressed in gastric cancer (GC) tissues and cells and closely associated with the overall survival of patients. **A** and **B**, The expression levels of miR-4290 in 60 GC tissues and the matched normal tissues were detected by RT-PCR assay. **C**, Kaplan-Meier analysis of the relationship between the expression levels of miR-4290 and patients' overall survival. **D**, RT-PCR assay was used to detect the expression of miR-4290 in GES-1, SGC7901, MKN45, and HGC-27 cell lines. Data are reported as means±SD (n=3). *P<0.05, **P<0.01 (ANOVA).

Compared to the adjacent non-tumor tissues, the miR-4290 expression level was significantly decreased in GC tissues (Figure 1A and B), and the low expression level of miR-4290 was closely associated with shorter survival in GC cases (Figure 1C). In addition, miR-4290 low expression predicted larger tumor size (P=0.001), high incidence of lymph node metastasis (P=0.002), and high TNM stage (P=0.002) (Table 1). Consistently, the expression of miR-4290 was reduced in GC cell lines HGC-27, SGC7901, and MKN45 compared to that in GES-1 cells (Figure 1D). These results suggested that miR-4290 was lowly expressed in GC tissues and cells, indicating its potential role in GC progression.

**Table 1:** Associations between miR-4290 expression and clinicopathological features in patients with gastric cancer.

Variables	miR-4290 expression		P value
	High (n=30)	Low (n=30)	
Age (years)			0.605
<60	15 (0.0%)	17 (6.7%)	
≥60	15 (0.0%)	13 (3.3%)	
Gender			0.796
Male	16 (3.3%)	15 (0.0%)	
Female	14 (6.7%)	15 (0.0%)	
Tumor size			0.001*
<3 cm	21 (0.0%)	8 (6.7%)	
≥3 cm	9 (0.0%)	22 (3.3%)	
Lymph node metastasis			0.002*
Absent (pN0)	19 (3.3%)	7 (3.3%)	
Present (pN+)	11 (6.7%)	23 (6.7%)	
TNM stage			0.002*
I-II	20 (6.7%)	8 (6.7%)	
III-IV	10 (3.3%)	22 (3.3%)	
HP infection			0.605
Yes	17 (6.7%)	15 (0.0%)	
No	13 (3.3%)	15 (0.0%)	

The median expression level of miR-4290 was used as the cut-off for high and low expression level. HP: *Helicobacter pylori*. *Statistically significant (chi-squared test).

### Upregulation of miR-4290 inhibited cell proliferation and induced cell apoptosis in GC

**Figure 2:**
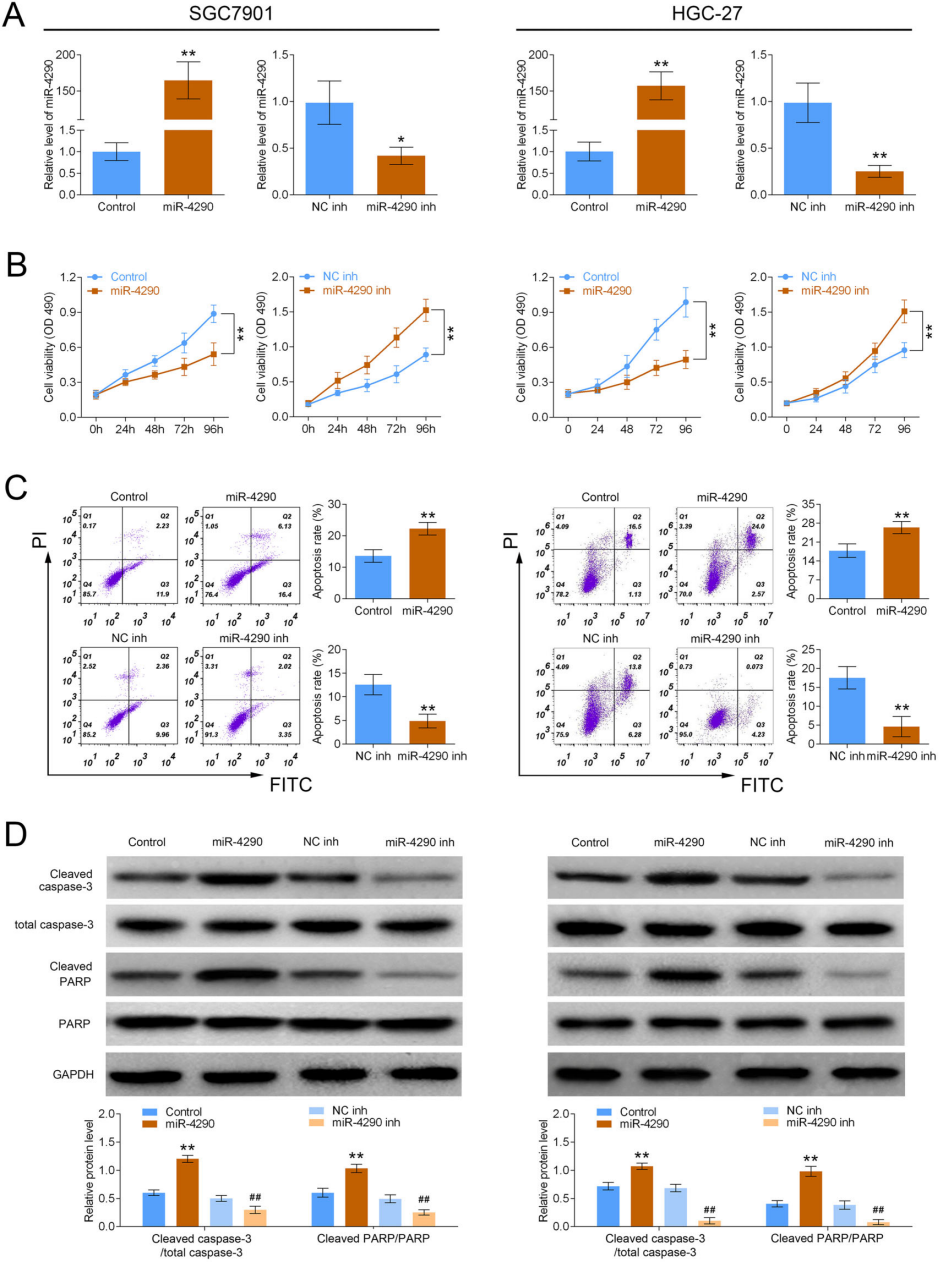
miR-4290 overexpression inhibited cell growth and induced cell apoptosis in gastric cancer (GC). **A**, The expression of miR-4290 was detected by RT-PCR assay after SGC7901 and HGC-27 cells were transfected with Control, miR-4290, Negative Control (NC) inh, or miR-4290 inh (n=3, *P<0.05, **P<0.01). **B**, CCK-8 assay was used to detect cell viability (n=3, **P<0.01). **C**, Flow cytometry assay was used to detect cell apoptosis (n=3, **P<0.01). **D**, The expressions of cleaved caspase-3, total caspase-3, cleaved PARP, and PARP were detected by western blotting assay in SGC7901 cells (left) and HGC-27 cells (right) (n=3, **P<0.05, compared with Control; ^##^P<0.05, compared with NC inh group). Data are reported as means±SD (ANOVA or *t*-test).

The gain/loss-of-function assays were carried out to explore the functions of miR-4290 in GC progression. Since miR-4290 showed higher expression in SGC7901 and HGC-27 cell lines than that in MKN45 cell lines, those were chosen for the following analysis. The miR-4290 expression was increased about 150-fold when SGC7901 and HGC-27 cells were transfected with miR-4290 mimics, whereas miR-4290 expression was reduced when cells were transfected with miR-4290-inhibitor (Figure 2A). Compared with the control group, miR-4290 overexpression significantly inhibited proliferation and promoted apoptosis of SGC7901 and HGC-27 cells, and knockdown of miR-4290 induced an increase in cell proliferation and a reduction in cell apoptosis rate (Figure 2B and C). Moreover, the expressions of cleaved caspase-3 and cleaved PARP were significantly increased when miR-4290 was overexpressed in SGC7901 and HGC-27 cells, and knockdown of miR-4290 caused opposite results (Figure 2D). These results demonstrated that miR-4290 functions as a tumor suppressor in GC.

### PDK1 was a target of miR-4290

**Figure 3:**
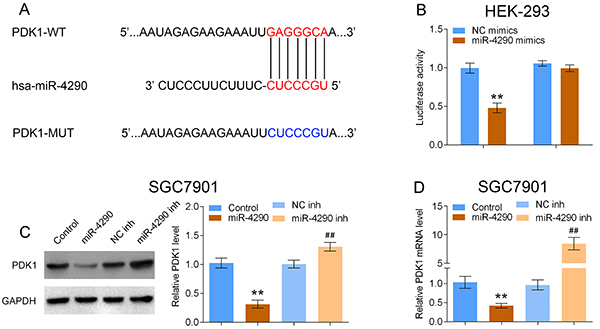
PDK1 was a direct target of miR-4290 in HEK-293 cells. **A**, The putative binding sites between miR-4290 and PDK1 and the mutated binding sites. WT, wide type; MUT, mutated type. **B**, The luciferase gene reporter assay was used to evaluate the relationship between miR-4290 and PDK1 after cell transfection with Negative Control (NC) mimics or miR-4290 mimics (n=3, **P<0.01). **C**, Western blotting analysis on the protein expression level of PDK1 after HEK-293 cells were transfected with Control, miR-4290, NC inh, or miR-4290 inh, and **D**, PDK1 RNA level (n=3, **P<0.05, compared with Control; ^##^P<0.05, compared with NC inh group). Data are reported as means±SD (ANOVA).

The relationship between PDK1 and miR-4290 in HEK-293 cells was evaluated. Figure 3A shows the putative binding sites between miR-4290 and the 3′UTR of PDK1. miR-4290 overexpression significantly reduced the luciferase activity of PDK1-WT, whereas this effect was abolished when the binding sites were mutated in HEK-293 cells (Figure 3B). In addition, the western blotting and RT-PCR results showed that miR-4290 could negatively regulate PDK1 expression at both protein (Figure 3C) and mRNA (Figure 3D) levels in SGC7901 cells. These above results suggested that PDK1 was a direct target of miR-4290.

### MiR-4290 inhibited glycolysis in GC cells via targeting PDK1

**Figure 4:**
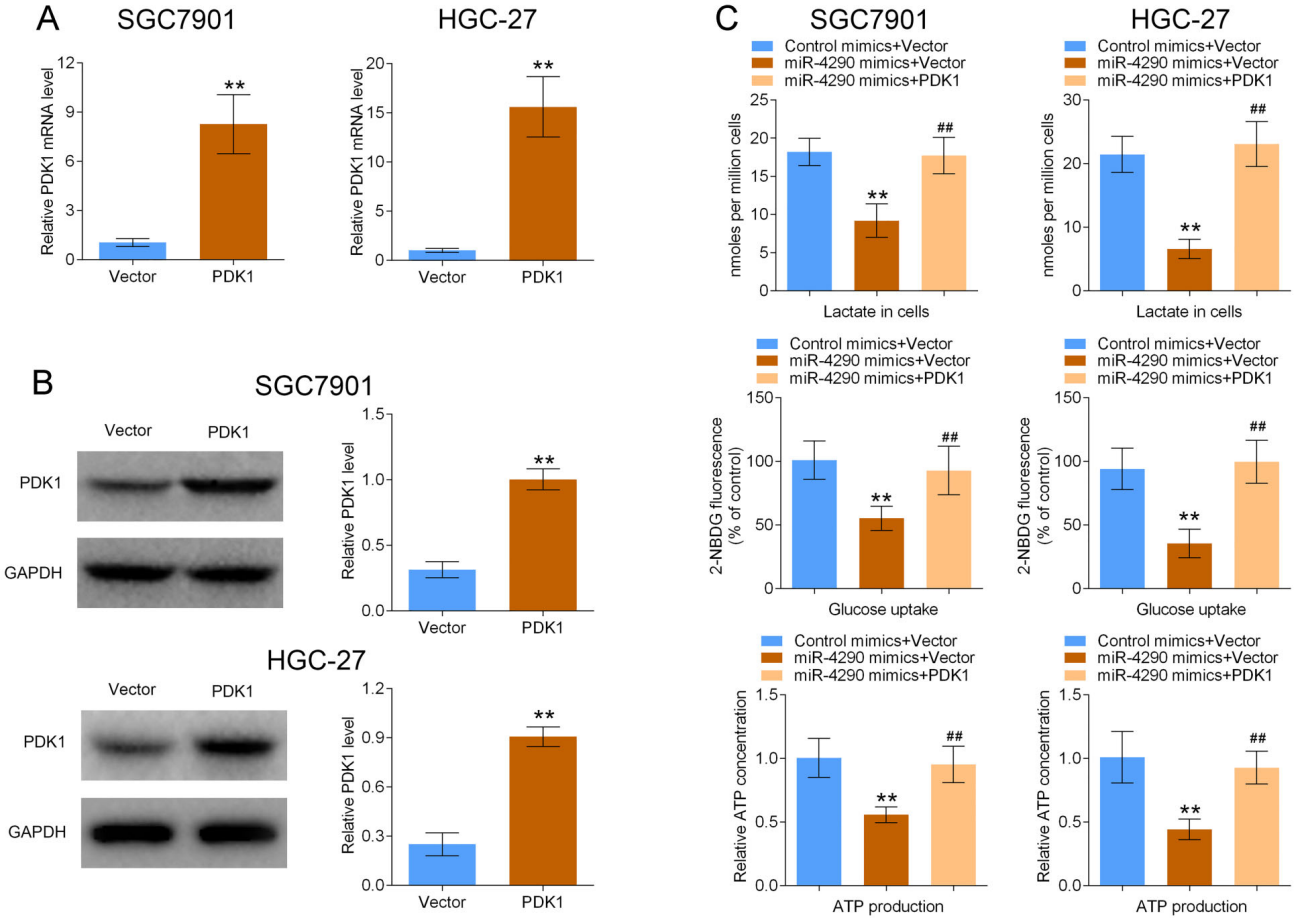
miR-4290 suppressed the glycolysis of gastric cancer cells via targeting PDK1. **A** and **B**, RT-PCR and western blotting assays were used to detect the mRNA and protein expression levels of PDK1 after SGC7901 and HGC-27 cells were transfected with Vector or PDK1 (n=3, **P<0.01). **C**, The glycolysis indicators, including lactate, glucose uptake, and ATP production were examined after SGC7901 and HGC-27 cells were transfected with Control mimics+Vector, miR-4290 mimics+Vector, and miR-4290 mimics+PDK1 (n=3, **P<0.01, compared with Control mimics+Vector group; ^##^P<0.01, compared with miR-4290 mimics+Vector group). Data are reported as means±SD (ANOVA or *t*-test).

PDK1 is reported to be closely implicated in glycolysis (22), and the effects of miR-4290/PDK1 axis on the glycolysis of GC cells were investigated. The expression levels of PDK1 at both mRNA and protein levels were significantly elevated when SGC7901 and HGC-27 cells were transfected with PDK1 overexpression vector (Figure 4A and B). Upregulation of miR-4290 induced a reduction in lactate content, glucose uptake, and ATP production, whereas these effects were increased when PDK1 was overexpressed in SGC7901 and HGC-27 cells (Figure 4C). These results demonstrated that miR-4290 inhibited the glycolysis of GC cells via targeting PDK1.

### MiR-4290 enhanced the cisplatin sensitivity of GC cells via targeting PDK1

**Figure 5:**
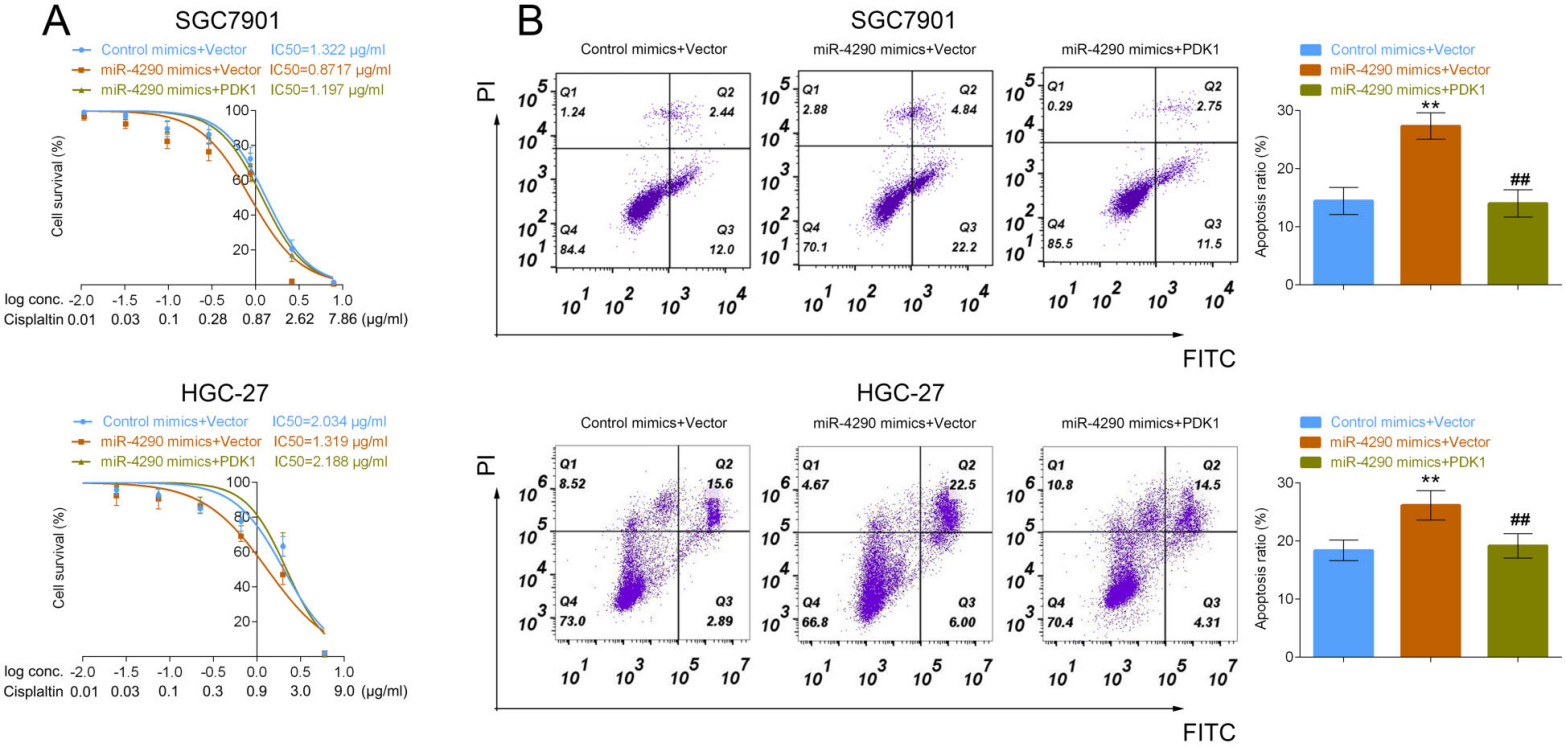
miR-4290 sensitized gastric cancer cells to cisplatin via downregulating PDK1 expression. **A**, CCK-8 assay was used to detect the concentration at 50% inhibition (IC50) value of SGC7901 and HGC-27 cells to cisplatin after the cells were transfected with Control mimics+Vector, miR-4290 mimics+Vector, and miR-4290 mimics+PDK1. **B**, Flow cytometry was used to detect cell apoptosis after SGC7901 and HGC-27 cells were transfected with Control mimics+Vector, miR-4290 mimics+Vector, and miR-4290 mimics+PDK1. **P<0.01, compared with Control mimics+Vector group; ^##^P<0.01, compared with miR-4290 mimics+Vector group. Data are reported as means±SD (ANOVA).

Then, we explored the effects of miR-4290/PDK1 axis on the sensitivity of GC cells to cisplatin. Overexpression of miR-4290 decreased the IC50 value of SGC7901 and HGC-27 cells to cisplatin, whereas the IC50 value was increased when PDK1 was overexpressed in SGC7901 and HGC-27 cells compared with the mimics+vector group (Figure 5A). In addition, PDK1 overexpression apparently abolished the increased apoptosis of SGC7901 and HGC-27 cells caused by miR-4290 mimics (Figure 5B). These results indicated that miR-4290 sensitized GC cells to cisplatin via downregulating PDK1 expression.

## Discussion

Chemotherapy in the form of neo-adjuvant or adjuvant therapy is a major treatment means for the majority of GC cases. Despite achievements in the development of new therapeutic drugs, cisplatin is still one of the most important drugs used for GC treatment (23). To enhance the chemosensitivity of GC cells to cisplatin, the roles of miR-4290/PDK1 axis in drug resistance were explored. The results demonstrated that miR-4290 could induce a significant repression in glycolysis to enhance the chemosensitivity of GC cells to cisplatin via directly binding to PDK1.

Several reports have identified that the deregulated miRNAs are implicated in the malignant phenotypic transformation of GC cells, including regulating cell survival, apoptosis, differentiation, and chemosensitivity. For instance, miR-181a was reported to be overexpressed in GC tissues, and its high expression was closely associated with GC patients' malignant progress and poor prognosis; silence of miR-181a significantly promoted GC cell apoptosis and suppressed cell proliferation, invasion, and metastasis, indicating that miR-181a functions as an oncogene in GC (24). In addition, miR-588 overexpression could cause obvious reductions in cell invasion, migration, and epithelial-mesenchymal transition (EMT), suggesting that miR-588 serves as a tumor suppressor in GC. All of these findings illustrate that miRNAs are implicated in GC progression. In the present study, we focused on the role of miR-4290 in GC progression, and the results demonstrated that miR-4290 showed a low expression pattern in GC tissues and cells, which predicated an advanced clinical process and shorter overall survival in GC cases. In addition, this study demonstrated for the first time, that miR-4290 functioned as a tumor suppressor in GC progression.

PDK1 was identified as a direct target of miR-4290 in GC cells via the luciferase gene reporter assay. PDK1 is an important glycolytic enzyme. Noticeably, evidence has shown that elevated uptake of glucose and enhanced glycolytic rates facilitate the unrestrained growth of cancer cells (5,25). Aerobic glycolysis, a shift from oxidative phosphorylation to glycolysis, and the incidental accumulation of lactate byproducts in the surrounding microenvironment accounts for the best-characterized alteration for cancer cell metabolism (26,27). In recent years, many reports have identified that the enhanced glycolysis is strongly implicated in drug resistance (28,29). In addition, the accelerated aerobic glycolysis obviously contributes to cisplatin resistance in GC (23). As PDK1 is an important glycolytic enzyme, we conjectured that miR-4290 might be involved in the regulation of glycolysis in GC cells via targeting PDK1. As expected, we observed that upregulation of miR-4290 induced obvious decreases in lactate content, glucose uptake, and ATP production, whereas these effects were increased when PDK1 was overexpressed in SGC7901 and HGC-27 cells, suggesting that miR-4290 could inhibit glycolysis via downregulating PDK1. Similarly, many miRNAs have been shown to play a crucial role in the glycometabolism of GC. For instance, miR-129-5p inhibited glucose metabolism and proliferation in GC cells via targeting SLC2A3 (solute carrier family 2 member 3), a glucose transporter (30). Via targeting PDHA1, miR-21-5p overexpression could significantly promote glycolysis and accelerate GC progression (31).

Accelerated glycolysis is strongly implicated in drug resistance (28,29), thus we explored the effects of miR-4290/PDK1 axis on the cisplatin sensitivity of GC cells. The results showed that miR-4290 overexpression reduced the IC50 of SGC7901 and HGC-27 cells to cisplatin and induced cell apoptosis, whereas PDK1 upregulation abolished this effect, indicating that miR-4290 improved the cisplatin sensitivity of GC cells via targeting PDK1 to inhibit glycolysis.

In conclusion, this study demonstrated that miR-4290 suppressed glycolysis and thereby enhanced the sensitivity of GC cells to cisplatin via targeting PDK1. Our study revealed that miR-4290/PDK1 might be a potent target to enhance the sensitivity of GC cells to cisplatin. However, one of the limitations of this study was that the role of miR-4290/PDK1 axis was not explored *in vivo*. We intend to explore the function of miR-4290/PDK1 axis in cisplatin resistance using the tumor-bearing models of GC.
